# The PrePex Device Is Unlikely to Achieve Cost-Savings Compared to the Forceps-Guided Method in Male Circumcision Programs in Sub-Saharan Africa

**DOI:** 10.1371/journal.pone.0053380

**Published:** 2013-01-21

**Authors:** Walter Obiero, Marisa R. Young, Robert C. Bailey

**Affiliations:** 1 Nyanza Reproductive Health Society, Kisumu, Kenya; 2 Department of Epidemiology and Biostatistics, University of Illinois at Chicago School of Public Health, Illinois, United States of America; Johns Hopkins University Bloomberg School of Public Health, United States of America

## Abstract

**Background:**

Male circumcision (MC) reduces the risk of heterosexual HIV acquisition in men by approximately 60%. MC programs for HIV prevention are currently being scaled-up in fourteen countries in sub-Saharan Africa. The current standard surgical technique for MC in many sub-Saharan African countries is the forceps-guided male circumcision (FGMC) method. The PrePex male circumcision (PMC) method could replace FGMC and potentially reduce MC programming costs. We compared the potential costs of introducing the PrePex device into MC programming to the cost of the forceps-guided method.

**Methods:**

Data were obtained from the Nyanza Reproductive Health Society (NRHS), an MC service delivery organization in Kenya, and from the Kenya Ministry of Health. Analyses are based on 48,265 MC procedures performed in four Districts in western Kenya from 2009 through 2011. Data were entered into the WHO/UNAIDS Decision Makers Program Planning Tool. The tool assesses direct and indirect costs of MC programming. Various sensitivity analyses were performed. Costs were discounted at an annual rate of 6% and are presented in United States Dollars.

**Results:**

Not including the costs of the PrePex device or referral costs for men with phimosis/tight foreskin, the costs of one MC surgery were $44.54–$49.02 and $54.52–$55.29 for PMC and FGMC, respectively.

**Conclusion:**

The PrePex device is unlikely to result in significant cost-savings in comparison to the forceps-guided method. MC programmers should target other aspects of the male circumcision minimum package for improved cost efficiency.

## Background

In 2007, WHO/UNAIDS recommended the promotion of male circumcision (MC) as an important additional HIV prevention strategy in areas of high HIV incidence, low MC prevalence, and with a predominantly heterosexual transmission epidemic [1]. This endorsement followed findings of randomized controlled trials (RCT) in Kenya [2], Uganda [3], and South Africa [4] that showed MC is approximately 60% protective against heterosexual HIV acquisition in men [5]. A recent population-level study in South Africa found age-standardized HIV prevalence was reduced from 12.5% to 9.3% among 15–49 year olds in a community three years following introduction of MC services [6]. Kenya, with an HIV prevalence of 6.3% among 15 to 49 year olds [7], and with several non-circumcising ethnic groups, was identified as one of the 14 priority countries in sub-Saharan Africa for scale-up of MC services for HIV prevention [8].

Modeling studies estimate that 80% MC uptake in priority countries with low MC prevalence would result in a 45% to 67% decline in HIV prevalence in those regions within a decade [9], [10]. Based on these findings and on expert recommendations, the governments of the 14 focal countries initiated plans to circumcise 20.34 million males between 2011 and 2015 and an additional 8.42 million males by 2025 with the aim of averting 3.36 million new infections by 2025 [11]. MC scale-up began in many of the priority countries of sub-Saharan Africa in 2008–2009 with the forceps-guided surgical method being the predominant method of choice. As of March 2012, approximately 1.54 million MC procedures had been performed in these countries [12]. The possibility that targets may not be attained in the desired period has led to calls for a review of current service delivery methods [13], [14].

Based on lessons learned during scale up of MC programs in several countries, including Kenya, various strategies have been developed to increase the efficiency of MC service provision. These include: task-shifting, task sharing, use of pre-packaged consumable supplies, use of multiple surgical bays, Rapid Results Initiatives and electrocautery use [15], [16], [17]. These innovations have had mixed success [13], [15]. Given the plan to provide MC services to over 20 million men over the next 3–5 years, data are urgently needed to inform rational programmatic decision-making that can lead to provision of high quality MC services at the lowest possible cost.

Some policy makers argue that a shift from FGMC to MC devices may reduce MC costs and increase technical efficiency of the procedure [18], [19]. Under consideration are MC devices that can be safely used by providers with minimal surgical training and who are working in resource-limited settings [19]. These devices may reduce intra-operative time and improve surgical outcomes. MC devices could potentially lead to cost-savings through the minimization of commodity inputs, increased provider productivity, increased demand for services and early resumption of activities of daily living [20], [21]. There are several adult male circumcision devices that are commercially available including: the Tara KLamp, the Alisklamp and the Shang Ring. There have been concerns about high rates of adverse events associated with some of these devices as well as need for skilled operators which may hinder their successful scale-up [22], [23].

MC implementers and policy makers are therefore actively considering an alternative technology, the PrePex device (Circ MedTech Ltd), which has recently undergone successful safety and efficacy trials in Rwanda, and has been approved for use there [24], [25]. The PrePex device comprises a placement ring, verification thread, inner ring, elastic ring and a reusable sizing plate [26]. There are five different ring sizes. After the penis is measured to ensure the correct size is applied, the device is affixed to the foreskin for a period of seven days. The rings occlude the preputial blood supply, leading to ischemic necrosis of the distal foreskin. The device is removed on the seventh post-procedure day, the necrotic foreskin is excised, and the wound is dressed [24]. Injected local anesthesia and suturing are not required during the procedure although use of a topical anesthetic (e.g., Lignocaine cream) during device placement is recommended [19], [25].

Our analysis compares the potential costs of introducing the PrePex device into routine MC services versus the current costs of MC service delivery under the forceps-guided method. This costing study is designed to contribute to discussions concerning the benefits of introducing the PrePex device into routine MC surgical practice for HIV prevention programming.

## Methods

This study assessed the projected per-MC cost of PrePex male circumcision (PMC) compared to forceps-guided male circumcision (FGMC). Costs under both scenarios were based on data from four Districts of Nyanza Province, Kenya for the period 2009 to 2011. A total of 48,265 MCs were provided during this time in the four Districts. The direct costs assessed include: drugs and supplies, personnel, and training. The indirect costs analyzed include: capital; maintenance and utilities; support personnel; and management/supervision.

Data for this study were extracted from routine program records compiled by the Nyanza Reproductive Health Society (NRHS). NRHS is a non-governmental organization that was founded in 2001 to be the lead Kenyan organization to conduct the randomized controlled trial of MC for HIV prevention in Kisumu, Kenya [2]. After completion of the RCT, NRHS transitioned into one of the key MC research, service provision and training organizations in Kenya. By the end of December 2011, NRHS had achieved over 186,000 circumcisions in the priority regions of the country, and had trained more than 1,800 healthcare workers of different cadres on the national curriculum of MC, which applies FGMC under local anesthesia [27]. We used data from the MC procedure database, program records, and financial reports.

Additional facility and district-level information was extracted from the Kenya Ministry of Health (MoH) Annual Operating Plan reports, which captures health sector resources and utilization rates across various districts in Kenya [28]. Clarification on some data was obtained from interviews with key managers at NRHS and with MoH officials.

We assumed that there would be minimal new requirements for in-country logistical management systems for PrePex, since the existing MC commodities supply chain infrastructure could be utilized. We assumed both PMC and FGMC would be provided in health care settings by two trained clinicians as recommended under current MoH regulations. However, for the PMC arm, we performed an additional analysis where the cost per circumcision was calculated under the assumption of one clinical provider assisted by a non-health care provider.

We assumed that PMC would reduce training time from ten days under FGMC to six days. Although the Rwandan program was able to provide training on PMC in three days [25], this would not be possible under Kenyan training protocols. Under the Kenyan National Guidelines for MC training, three days of the course are didactic sessions covering the following topics: MC and HIV; linking MC to other reproductive health services; client education/counseling/consenting; screening/STI management; overview of available surgical techniques; post-operative care; infection prevention; and monitoring and evaluation (M&E)/quality assurance (QA) [27]. The bulk of the theoretical curriculum would be unchanged under PMC. In addition, although PMC is easier to learn and a faster procedure, in Kenya our experience is that at least three days would be required to recruit a sufficient number of clients (e.g., 10–20 men for each of several trainees) to achieve competency in the PrePex method of circumcision.

We assumed PMC would: require no infrastructure investments (e.g., renovations of operating rooms), require one half of the personnel costs for mobilization of clients, reduce intra-operative time from 15 to five minutes, require one half the FGMC full-time equivalents of supervision for quality assurance, and obviate sterilization costs and the need for routine clinical supplies (e.g., blood pressure cuff, weighing scale, and thermometer). We assumed there would be minimal impact on delivery of other components of the MC minimum package (e.g., STI management, M&E, and HIV testing and counseling).

We assumed that all clients undergoing PMC would need at least one post-procedure day seven visit for excision of necrotic foreskin tissue and device removal [24]. Recommendations for number of post-device removal visits have not been issued, though additional visits are unlikely to be necessary. For the FGMC analysis, we used a post-operative return rate of 41% to reflect current experience and 60% to reflect the baseline recommendation for Kenya [27].

We performed separate PMC analyses under the assumption that 0%, 5%, 10% and 15% of clients would be excluded due to phimosis or tight foreskin [19], [24], [25]. This assumption increases the PMC cost by assuming fewer clients are circumcised, but does not account for referral costs, personnel, or commodity costs needed to circumcise the excluded clients. We supposed PMC service providers would be trained on the PrePex method only. The FGMC method can be modified with the addition of a dorsal slit for clients with phimosis or tight foreskin.

For FGMC, we performed separate analyses for the observed adverse event rate of 0.9% [13] and a rate of 2.17% – assuming clients not returning for review experienced the same rate of AEs as those who returned. We used observed adverse event types for cost computations (i.e., 0.5% of clients had cases of infection requiring surgical toilet, antibiotics and two additional post-surgical visits). For PMC, we used AE rates of 0.5% and 1% and assumed all AEs were cases of swelling that required an additional visit for consultation and reassurance (which adds personnel costs but no consumable costs). The true AE rate for PMC is not known, though a small safety study in Rwanda detected one swelling AE in 50 participants [24]. A larger study which included 150 PrePex procedures found no device-related AEs and four non-device related AEs [25]. We did not include the price of the PrePex device itself, because its retail price has not been determined.

Data were entered into the Decision Makers Program Planning Tool (DMPPT) [29], a Microsoft Excel program that has been widely used to calculate the costs and impacts of various MC programs in sub-Saharan Africa [11], [14], [30]. The data entry tool was modified to reflect consumables currently used in the Kenyan MC program (e.g., the antibiotic cloxacillin was replaced with ceftriaxone). In addition, some personnel categories were also altered (e.g., the outpatient department nurse was replaced with a clinical officer). The reported costs were discounted at an annual rate of 6% to allow for future comparisons. The costs are reported in United States Dollars (USD) at the current Central Bank of Kenya exchange rate of 85 Kenya Shillings to one USD.

This paper used data that were collected as part of routine program delivery. MC clients could not be identified – either directly or through identifiers linked to individuals. Consistent with Human Research Protection Policy {45CFR46.101(b)(4)}, we did not seek IRB approval.

## Results

The study assessed direct and indirect costs of the PrePex MC (PMC) method compared to the forceps-guided MC method (FGMC). The costs of one MC surgery were $44.54 and $55.29 for PMC and FGMC, respectively (see Table 1). The PMC cost assumes 0% of clients with phimosis/tight foreskin, AE rate of 0.5%, and one clinician performing the procedure assisted by a non-clinician (best-case scenario). The PMC estimate does not include the cost of the PrePex device, itself. The FGMC cost assumes 60% review rate and 2.17% AE rate (worst-case scenario). The cost of the forceps-guided method declined by less than a dollar (to $54.52) when the review rate and AE rate were lowered to the observed rates (41% and 0.9%, respectively). The cost of PMC increased by over three dollars (to $47.76) when prevalence of phimosis was assumed to be 10% (and therefore number of clients circumcised were reduced by 10%) – though the PrePex device and referral surgery costs are not included in this figure. Using two clinicians added $1.26 to the cost of a PMC procedure.

**Table 1:** Cost in USD (%) of one MC surgery using PrePex and forceps-guided method under various assumptions.

		Assumptions	Direct Costs				Indirect costs				*Subtotal (Direct Costs)*	*Subtotal* *(Indirect Costs)*	Total
			Consumable supplies	Non-consumable supplies	Direct Personnel	Training	Capital	Maintenanceand utility	Support personnel	Management and supervision			
Forcepsguided	1	F/U = 60% AE rate = 2.2%	9.35 (17)	6.71 (12)	10.72 (19)	0.97 (2)	2.57 (5)	3.47 (6)	10.78 (20)	10.72 (19)	*27.75 (50)*	*27.54 (50)*	55.29
	2	F/U = 41% AE rate = 0.9%	8.91 (16)	6.51 (12)	10.59 (19)	0.97 (2)	2.57 (5)	3.47 (6)	10.78 (20)	10.72 (20)	*26.98 (49)*	*27.54 (51)*	54.52
PrePex	3	phimosis = 0% 2clinicians AE rate = 0.5%	5.32 (12)	5.45 (12)	8.03 (18)	0.65 (1)	2.52 (6)	3.47 (8)	9.64 (21)	10.72 (23)	*19.44 (42)*	*26.35 (58)*	45.79
	4	phimosis = 10% 2clinicians AE rate = 0.5%	5.34 (11)	5.48 (11)	8.06 (16)	0.72 (1)	2.82 (6)	3.87 (8)	10.77 (22)	11.97 (24)	*19.60 (40)*	*29.43 (60)*	49.02
	5	phimosis = 0% 1 clinician, 1 non-clinician AE rate = 0.5%	5.32 (12)	5.45 (12)	6.77 (15)	0.65 (1)	2.52 (6)	3.47 (8)	9.64 (22)	10.72 (24)	*18.18 (41)*	*26.35 (59)*	44.54
	6	phimosis = 10% 1clinician, 1 non-clinicianAE rate = 0.5%	5.34 (11)	5.48 (11)	6.80 (14)	0.72 (2)	2.82 (6)	3.87 (8)	10.77 (23)	11.97 (25)	*18.34 (38)*	*29.43 (62)*	47.76

AE, Adverse Event; F/U, follow-up; not all percentages add to 100 due to rounding.

**Consumable Supplies.** *Both methods:* non-sterile gloves, alcohol hand rub, antiseptic soap, providone antiseptic solution, diclofenac {analgesic} tabs, paraffin gauze {Vaseline gauze}, gauze swabs, cohesive bandage. *PMC:* Lignocaine cream {local anesthesia cream}. *FGMC:* sterile gloves, face mask, Bupivacaine, Lignocaine, Needles (21 gauge and 19 gauge), 10 ml syringe, surgical blade (size 10), chromic catgut suture 3/0.

**Non-consumable supplies.** *Both methods:* client underpants. *FGMC:* weighing scale, blood pressure cuff, thermometer, surgical scrubs, shoe cover, sterile drape, center 'o', circumcision surgical tray (gallipot Kocher clamp mosquito artery forceps, blade holder, kidney dish, Dunhill artery forceps, tissue scissors, suture scissors, Adson forceps, needle holder, sponge holding forceps), emergency tray supplies. *PMC:* dressing tray (kidney dish, gallipot, sponge holding forceps, scissors).

**Direct Personnel:** *Both methods:* counselor, hygiene officer. *FGMC:* Clinical officer, nurse, consultant urologist (for AEs). *PMC:* Nurse/nurse pair or nurse/nurse aide pair.

**Training:** *Both methods*: 3 days of theory. *FGMC:* 7 days practicum PMC: 3 days practicum; costs include staff time, lunch/refreshments for participants and trainers, stationery, and MC manual.

**Capital:** *Both methods:* autoclave (PMC assumed to use 1/2 that of FGMC), surgical couch, cell phones, incinerator, office furniture, vehicles, facility space, generator, surgical equipment (mayo tray, trolley, waste bin).

**Maintenance and Utility:** *Both methods:* internet, office rent, electricity (power), water, vehicle costs (maintenance, insurance, and fuel). *FGMC:* facility renovations.

**Support Personnel:** *Both methods:* MOH supervision, quality assurance/quality improvement (QA/QI) team, training team, department managers and staff (human resources, transport, finance, administration, stores, information technology, data, mobilization). *PMC:* MOH supervision, mobilization team, QA/QI team and training team assumed to require 1/2 the full-time equivalents of FGMC.

**Management and Supervision:** *Both methods:* senior management team salaries, travel expenses for circumcision camps and management supervision.

The categories that account for the largest proportion of costs under both programs are direct personnel, support personnel, and management/supervision. These three categories account for approximately 60% of the total cost under both PMC and FGMC. There was minimal difference between FGMC and PMC in indirect costs. Direct personnel costs associated with the FGMC method (average $10.66) were higher than for PMC (average $7.42) due to longer operating time and higher assumption of adverse events. Consumable supply costs were also higher under FGMC than PMC ($9.13 vs. $5.33 on average), though the addition of the PrePex device would increase this cost. Although FGMC consumable supply costs are higher than PMC on the procedure day, the PMC method necessitates 100% day 7 review rates for device removal. The removal visits require additional consumable supplies e.g., clean gloves, handrub, gauze, scalpel, etc. Similarly, PMC requires fewer non-consumable instruments than FGMC, but costs in this category were estimated to be only moderately lower under PMC than FGMC ($5.47 vs. $6.61 on average), since 100% of PrePex clients are assumed to return for a 7 day post-procedure visit and utilization of certain supplies (e.g., dressing tray for device placement/removal) are amortized over each visit. Although potentially not required by all programs, client underpants (cost $0.59 each), are used in our program and were included under both methods. Other costs are shown in Table 1.

## Discussion

The cost of one FGMC in Kenya was higher than PMC – $54.52–$55.29 versus $44.54–$49.02– though the PMC cost does not include the cost of the PrePex device or referral of clients who are ineligible for PMC due to phimosis/tight foreskin. These clients would need to be circumcised using conventional surgical methods, such as FGMC modified with addition of a dorsal slit. Our figures fall between previous MC costing estimates by Kioko [30] and Njeuhmeli, *et al.* [11] who calculated a per FGMC cost in Kenya of $32.04 (weighted average cost per MC at fixed-sites) and $74.89, respectively.

The cost categories that were most sensitive to MC procedure method were consumable supplies and direct personnel. Other client-oriented (e.g., counseling, STI screening and management) and program running (e.g., vehicles, utilities, and supervision) were insensitive to method of MC. Under the PrePex method of MC, it is necessary to review the client one week after device placement to remove the ring and excise necrotic foreskin. Additional visits may be required for bandage removal or wound healing assessment, though these costs were not included in the analysis. Higher review rates under either method would represent an additional opportunity for risk-reduction counseling, although the public health impact of additional review visits may be minimal [31], [32].

Several of our assumptions likely resulted in the underestimation of PMC costs. We used low AE rates for PMC (0.5% and 1%) and included only potential cases of swelling [24]. Studies from other MC devices have detected other common MC-related AEs, including device detachment, wound dehiscence, bleeding, infection, and pain [20], [23], [33]. Although we assumed that only one size of the device would be required, there are five different adult PrePex ring sizes. Logistical management systems necessary to deliver and maintain more than one device size to various facilities would likely add to the PMC cost [34]. Finally, a recent study found healing time under PMC was approximately two weeks longer than under a standard surgical method [25] which could potentially lead to increased post-procedure visits and a longer recommended abstinence period.

The use of the PrePex device is contraindicated in 5–18% of all clients due to existence of phimosis or tight foreskin and these clients will require MC surgery using other techniques, such as FGMC modified with addition of a small dorsal slit [19], [20], [25], [27]. Additionally, approximately half (53%) of MCs included in this analysis were of 10 to 17 year old clients. Currently, PrePex has only been studied in HIV negative clients aged 18 and above [19]. Therefore, alternative mechanisms would be needed to circumcise clients who are ineligible for PMC. These mechanisms would likely increase the cost of PMC substantially through need for additional training of providers or client referral costs.

Documented barriers to MC include: fear of adverse events (including pain), lengthy post-surgical abstinence period and delayed resumption of work [35], [36]. Several device studies have indicated good client satisfaction due to improved cosmetic outcome, faster return to work and low AE rate [20], [25]. The analysis did not assess the potential cost-savings that may accrue due to such positive spillover effects on demand creation following the introduction of PMC, though we did assume 50% lower mobilization costs. This assumption decreased the cost of PMC by approximately $0.40 per procedure.

A challenge of the forceps guided surgical technique has been difficulty in standardizing the procedure. There is a lack of consistency in the amount of local anesthesia administered and variability in cosmetic outcome. The PrePex device has the potential to minimize these provider-associated variations, maximize the benefits of task-shifting and lead to cost savings. The paper did not assess these potential cost-savings.

This study has several limitations. Costing data were obtained from a single region in Kenya and costs may be different in other regions in the country or in other nations. We did not include supply chain management costs or waste disposal costs, which have been found to add substantially to the per-MC cost [34]. The calculations of the PrePex costs were largely hypothetical, but based on information available from published studies and from observed program costs, many of which would be fixed regardless of MC method. We could not include the cost of the device itself because the manufacturer has not set a price, nor can we know the costs of distribution. To our knowledge, this is the first paper to directly compare costs of an MC device to those of standard surgical practice. We based costs on a large number of circumcisions conducted over a two-year time period.

### Conclusion

The PrePex device is unlikely to result in significant cost-savings in comparison to the forceps-guided method. Indeed, depending on the price of the device, PrePex may well cost significantly more than the forceps-guided method. Because the surgery is only one component of a comprehensive VMMC program, MC programmers should scrutinize other aspects of the male circumcision minimum package to achieve greater cost efficiencies.
